# Risk factors for retear of arthroscopic rotator cuff repair using triple-row technique

**DOI:** 10.1016/j.jseint.2025.101412

**Published:** 2025-12-02

**Authors:** Ryosuke Takahashi, Ryosuke Sagami, Yohei Harada, Yukihiro Kajita

**Affiliations:** a Department of Orthopaedic Surgery, Ichinomiya Nishi Hospital, Ichinomiya, Japan; b Department of Orthopaedic Surgery, Hiroshima University, Hiroshima, Japan

**Keywords:** Arthroscopic rotator cuff repair, Triple-row technique, Retear, Range of motion, Clinical outcome, Infraspinatus tendon

## Abstract

**Background:**

The triple-row (TR) technique enhances tendon-to-bone contact and repair integrity in arthroscopic rotator cuff repair (ARCR). However, limited studies have evaluated the clinical outcomes and risk factors for retears following this technique. This study aimed to compare clinical outcomes between retear and nonretear groups after ARCR using the TR technique and identify risk factors for retear.

**Methods:**

Patients who underwent ARCR using the TR technique with at least 24 months follow-up were enrolled and categorized into retear and nonretear groups. Clinical outcomes, including range of motion, visual analog scale for pain, Constant Shoulder (CS), and University of California Los Angeles (UCLA) score, were evaluated at 3, 6, and 24 months postoperatively. The retear rate and tendon integrity were assessed at 3, 6, and 24 months follow-up using magnetic resonance imaging, and risk factors for retear were analyzed.

**Results:**

Of 181 patients enrolled, 20 (11%) had retears and 161 (89%) had no retears. The retear group was significantly older (*P* = .037) and had more large or massive tears (*P* = .044), larger tear sizes on magnetic resonance imaging (coronal plane: 29.9 ± 14.9 mm vs. 21.6 ± 12 mm, *P* = .005; sagittal plane: 23.7 ± 12.1 mm vs. 17.9 ± 9.4 mm, *P* = .013), and greater fatty infiltration (*P* < .001). Both groups achieved good clinical outcomes, but the retear group showed significantly lower CS and UCLA scores at 6 and 24 months (CS score: 72.4 ± 9 point vs. 78.6 ± 8.6 point at 6 months [*P* = .004] and 81.2 ± 11.1 point vs. 86.3 ± 9.6 point at 24 months [*P* = .032]; UCLA score: 25 ± 2.6 point vs. 27.3 ± 3.5 point at 6 months [*P* = .005], 28.7 ± 4.6 point vs. 30.9 ± 3.7 point at 24 months [*P* = .018]) and higher visual analog scale scores at 24 months (21.5 ± 12 vs. 7.8 ± 7.7, *P* = .021). Fatty infiltration of the infraspinatus was identified as an independent risk factor for retears. Patients with Goutallier grade ≥3 had significantly inferior external rotation at 3 and 6 months (26 ± 25.1° vs. 44.7 ± 11.4°, *P* = .032, 35 ± 28.3° vs. 53 ± 9°, *P* = .038) and tended to show inferior outcomes overall.

**Conclusion:**

ARCR using the TR technique provides favorable clinical outcomes, even in cases with retears. However, patients with retears—especially those with large-to-massive tears or severe infraspinatus fatty infiltration—showed inferior outcomes.

Arthroscopic rotator cuff repair (ARCR) is a widely practiced and accepted treatment for rotator cuff tears. While most patients experience satisfactory clinical outcomes after ARCR with the advancement of various arthroscopic techniques, such as double-row and suture bridge (SB) techniques, the rate of retears remains high (10%-30%).^5^^,^^8^^,^^9^^,^^20^^,^^23^

The triple-row (TR) technique was first described by Ostrander and McKinney in 2012.^17^ This technique enhances the contact area and pressure on the rotator cuff footprint compared to the SB technique.^10^^,^^25^ This technique modifies the standard SB technique by incorporating an additional middle-row anchor between the medial and lateral rows on the lateral footprint of the greater tubercle. Sutures were first passed through the torn end of the rotator cuff before performing the standard SB technique. The TR technique has the potential to improve the reproducibility of the planned repair design compared to the standard SB technique.

Although favorable clinical outcomes of the TR technique have been reported, limited studies have specifically examined the clinical outcomes of patients with retears or identified the risk factors for retear following this procedure.^10^^,^^25^ Therefore, this study aimed to compare clinical outcomes between patients with a retear and those with an intact repair following ARCR using the TR technique and to identify risk factors associated with retear in this procedure. We hypothesized that ARCR using the TR technique achieves good clinical outcomes even in cases of retear; however, outcomes in retear cases would be inferior to those in healed cases.

## Materials and methods

### Study design

This retrospective study was approved by the institutional review board of our hospital. We identified 262 patients with full-thickness rotator cuff tears who underwent ARCR using the TR technique performed by a single surgeon between January 2020 and February 2023. Patients were eligible for surgery if they had a primary rotator cuff tear and had not responded to conservative treatment for at least 6 months. We enrolled patients who met the following inclusion criteria: presence of complete rotator cuff tears, including the supraspinatus tendon, as verified by preoperative magnetic resonance imaging (MRI); patients who underwent complete rotator cuff repair; and patients who underwent follow-up for at least 24 months after ARCR, with an evaluation of successful repair using MRI. Patients were excluded if they had isolated subscapularis tears, irreparable rotator cuff tears, revision surgery, or partial rotator cuff repair.

Rotator cuff tear size was evaluated using MRI by measuring the longitudinal and transverse dimensions of the tears along the oblique coronal and sagittal planes. The tear type was categorized according to size as small (<1 cm), medium (1-3 cm), large (3-5 cm), or massive (>5 cm) according to the classification reported by Cofield et al.^4^

**Figure 1:**
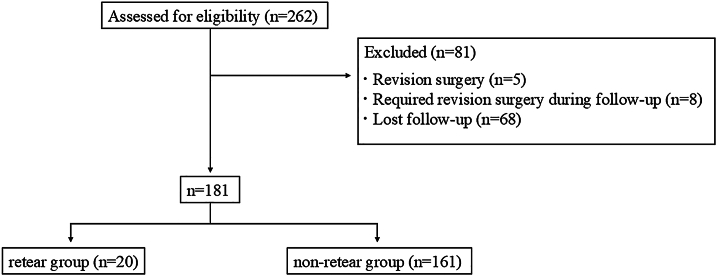
Study design flow diagram.

A total of 262 ARCR procedures were performed during the study period. After excluding 81 patients, 181 were included in this study (Fig. 1).

### Surgical technique

**Figure 2:**
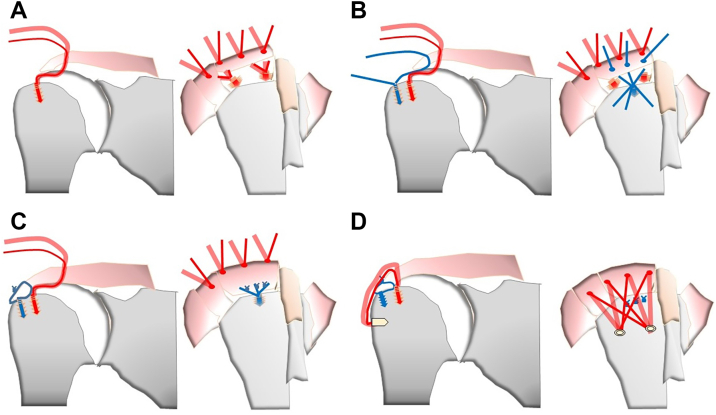
The scheme of repair of triple-row technique (right shoulder). Insertion of the medial row anchor (**A**). Insertion of the middle row anchor (**B**). Sliding knot to tie the suture of the middle row anchor (**C**). Fixation of the medial row anchor suture using the lateral row anchor (**D**).

All surgical procedures were performed under general anesthesia in a beach chair position by a single experienced surgeon (R.T.). The details of the TR technique are shown in Figure 2. Sufficient joint mobilization was performed around the rotator cuff before repair, and a medial row anchor (HEALICOIL RG; Smith & Nephew Endoscopy, Andover, MA, USA) was inserted medially into the humeral footprint and rotator cuff tear. The sutures of the medial row anchor were passed through the rotator cuff 10–15 mm medially to the lateral edge of the torn tendon (Fig. 2, *A*). No knot tying was performed on the rotator cuff. After the sutures of the medial anchors were passed through the rotator cuff, a middle-row anchor (Healix BR; DePuy-Mitek, Raynham, MA, USA) was inserted into the lateral edge of the footprint (Fig. 2, *B*). Sutures were passed through the torn tendon of the rotator cuff with a simple stitch and tied using a sliding knot (Fig. 2, *C*). Finally, sutures from the medial anchors were retrieved and secured by inserting lateral row anchor (Quatro Link knotless; Zimmer Biomet, Warsaw, IN, USA) into the lateral aspect of the greater tuberosity (Fig. 2, *D*). The number of anchors was determined based on tear size and repair configuration.

All patients underwent the same postoperative rehabilitation program. The affected shoulder was immobilized using an abduction brace for 4 weeks in cases of small-to-medium tears and for 6 weeks in large-to-massive tears (Global Sling; COSMOS, Sapporo, Japan). Elbow, wrist, and finger exercises were initiated immediately after the surgery. Passive forward flexion exercises were initiated on the first postoperative day, with no restrictions on the patient's passive range of motion (ROM). Active-assisted motion exercises were initiated at 4 weeks for small-to-medium tears and at 6 weeks for large-to-massive tears postoperatively. Active motion was allowed at 6 weeks for small-to-medium tears and at 8 weeks for large-to-massive tears postoperatively. A strengthening exercise program was permitted at 8 weeks for small-to-medium tears and at 12 weeks for large-to-massive tears postoperatively. Rehabilitation was performed at least 3 months after surgery with the assistance of a physical therapist. Full return to sports or heavy labor was allowed after 6 months.

### Clinical outcomes

Clinical outcomes were evaluated between the retear and nonretears groups. The following patient demographics were evaluated: sex, age, tear size, fatty infiltration of the rotator cuff using the Goutallier classification,^6^ ROM, visual analog scale for pain, Constant Shoulder (CS) score, and University of California Los Angeles (UCLA) score at 3, 6, and 24 months postoperatively. Moreover, we investigated the risk factors for retear using multiple logistic regression analysis. Internal rotation was defined as the highest vertebral body that the patient could reach using the thumb of the affected arm. Internal rotation was scored as follows: above Th12, 6 points; above L5, 4 points; on the buttocks, 2 points; and below the buttocks, 0 points. The scoring system was based on the Japanese Orthopedic Association shoulder score.^23^ The integrity of the repaired tendon was assessed at 3, 6, and 24 months follow-up using MRI. Repair integrity after ARCR was classified into five categories according to the Sugaya classification using oblique coronal, oblique sagittal, and transverse views of T2-weighted images.^22^ Types 4 and 5 were considered retears using this classification system.

### Statistical analysis

The chi-square test was performed to compare categorical variables such as sex ratio, affected side, tear type according to size, and number of torn tendons between the groups. Student's *t*-test was performed to compare age, ROM, functional scores, tear size, and fatty infiltration between the groups, while a paired t-test was performed to compare these variables between two consecutive periods in each group. All statistical analyses were performed using Statistical Package for the Social Sciences (SPSS) (version 18.0; SPSS Inc., Chicago, IL, USA). Statistical significance was set at a *P* value of < .05. Factors for retear were analyzed by using logistic regression models. Multivariate logistic regression analysis (forward stepwise (likelihood ratio)) was performed using variables that were significant in univariate analysis.

## Results

A total of 262 ARCR procedures were performed during the study period. After excluding 81 patients, 181 were included in this study (Fig. 1). The mean age of the included patients was 63.5 ± 10.1 years and the mean follow-up duration was 30.3 ± 8.9 months. Of the 181 patients who underwent ARCR using the TR technique, 20 (11%) had a retear on MRI 24 months postoperatively, while 161 (89%) had no retear.

The patient demographic characteristics are summarized in Table I. The mean age of the retear group was significantly higher than that of the nonretear group (67.2 ± 10.8 years vs. 63.1 ± 10 years, *P* = .037). In the retear group, the number of large and massive tear were significantly more frequent (*P* = .044), and the preoperative tear size on MRI was significantly larger in both the coronal and sagittal planes than in the nonretear group (coronal plane: 29.9 ± 14.9 mm vs. 21.6 ± 12 mm, *P* = .005; sagittal plane: 23.7 ± 12.1 mm vs. 17.9 ± 9.4 mm, *P* = .013). In addition, preoperative fatty infiltration of the rotator cuff was significantly more severe in the retear group than in the nonretear group (*P* < .001). Both groups achieved good clinical outcomes at 24 months postoperatively (Table II). At 3 months, the retear group had greater flexion than the nonretear group (146.5 ± 31°, 135.5 ± 21.9°, *P* = .046). At 6 and 24 months, the retear group showed significantly lower CS and UCLA score (CS score: 72.4 ± 9 point vs. 78.6 ± 8.6 point at 6 months [*P* = .004] and 81.2 ± 11.1 point vs. 86.3 ± 9.6 point at 24 months [*P* = .032]; UCLA score: 25 ± 2.6 point vs. 27.3 ± 3.5 point at 6 months [*P* = .005], 28.7 ± 4.6 point vs. 30.9 ± 3.7 point at 24 months [*P* = .018]). At 24 months, the retear group showed a significantly higher visual analog scale score (21.5 ± 12 vs. 7.8 ± 7.7, *P* = .021). Moreover, the retear group showed more severe fatty infiltration of the rotator cuff, which persisted at 3, 6, and 24 months (*P* < .001). Multiple logistic regression analysis revealed the fatty infiltration of the infraspinatus (ISP) tendon as independent risk factor for retear of this procedure (odds ratio = 4.5, 95% confidence interval: 2.3-8.9, *P* < .001) (Table III). When clinical outcomes were compared according to the severity of fatty infiltration of ISP, patients with a Goutallier grade of 3 or higher had significantly inferior external rotation at 3 and 6 months postoperatively than those with a grade of 2 or lower (26 ± 25.1° vs. 44.7 ± 11.4° at 3 months, *P* = .032, 35 ± 28.3° vs. 53 ± 9° at 6 months, *P* = .038). Although there were no statistically significant differences in other ranges of motion or clinical scores, patients with a Goutallier grade of 3 or higher tended to show worse outcomes (Table IV).

**Table I:** Patient's demographics at baseline.

Variables	Retear group	Nonretear group	*P* value
Number of shoulders	20	161	
Male/female (n)	10/10	108/53	.142
Age (yr)	67.2 ± 10.8	63.1 ± 10	.037
Affected side (right/left) (n)	18/2	154/7	.260
The presence of diabetes mellitus (n)	5	32	.565
Duration of symptoms before surgery (mo)	15.1 ± 17.2	12.8 ± 19.2	.610
Body mass index (kg/m^2^)	23.9 ± 3.7	24.6 ± 3.8	.435
Tear type according to size (n)			.044
Small	2	40	
Medium	8	82	
Large	7	34	
Massive	3	5	
Tear size∗			
Amount of retraction (cm)	29.9 ± 14.9	21.6 ± 12	.005
Anteroposterior dimension (cm)	23.7 ± 12.1	17.9 ± 9.4	.013
Fatty infiltration†			
SSC (0/1/2/3/4)	0/8/11/1/0	25/112/24/0/0	<.001
SSP (0/1/2/3/4)	0/0/5/13/2	0/28/94/36/3	<.001
ISP (0/1/2/3/4)	0/8/7/5/0	20/111/28/2/0	<.001
TM (0/1/2/3/4)	2/10/5/1/2	69/75/11/4/2	<.001
Preoperative findings			
ROM			
Forward flexion (°)	156 ± 19.3	144.2 ± 31.1	.099
ER (°)	53 ± 10.1	48.7 ± 14	.182
IR (point)	4.7 ± 1.0	4.2 ± 1.5	.154
CS score (point)	56.8 ± 15.1	53.8 ± 13.9	.396
UCLA score (point)	19.2 ± 3.9	18.2 ± 3.7	.325
VAS	47.4 ± 18.3	51.1 ± 23.2	.654

*SSC*, subscapularis; *SSP*, supraspinatus; *ISP*, infraspinatus; *TM*, teres minor; *ER*, external rotation; *IR*, internal rotation; *CS* score; Constant Shoulder score; *UCLA score*; University of California Los Angeles score; *VAS*, visual analog scale; *ROM*, range of motion.

∗ Tear size was measured intraoperatively after débridement of the degenerated tendon edges. Tear size in the AP, dimension was measured at the lateral edge of the footprint, and the amount of retraction was estimated by the distance from the apex of the tear to the footprint.

† Fatty infiltration was graded according to the criteria established by Goutallier et al.

**Table II:** Comparison of postoperative findings.

Variables	Retear group	Nonretear group	*P* value
ROM			
Forward flexion (°)			
POD 3M	146.5 ± 31	135.5 ± 21.9	.046
POD 6M	160.5 ± 19.3	157.9 ± 18	.550
POD 24M	164.0 ± 17.3	164.9 ± 13.9	.790
ER (°)			
POD 3M	40 ± 17.2	35.9 ± 13.7	.228
POD 6M	48.5 ± 17.1	48.7 ± 12.7	.942
POD 24M	53.0 ± 18.7	54.2 ± 11.2	.673
IR (point)			
POD 3M	4.0 ± 1.2	3.6 ± 1.3	.161
POD 6M	4.6 ± 0.9	4.6 ± 1.0	.971
POD 24M	5.2 ± 1.0	5.4 ± 1.0	.531
CS score (point)			
POD 3M	69.2 ± 6.4	71.7 ± 7.7	.329
POD 6M	72.4 ± 9.0	78.6 ± 8.6	.004
POD 24M	81.2 ± 11.1	86.3 ± 9.6	.032
UCLA score (point)			
POD 3M	23.7 ± 2.3	24.1 ± 321	.676
POD 6M	25.0 ± 2.6	27.3 ± 3.5	.005
POD 24M	28.7 ± 4.6	30.9 ± 3.7	.018
VAS			
POD 3M	23.5 ± 23.3	23.7 ± 19.4	.987
POD 6M	28 ± 31.1	13.3 ± 11.1	.115
POD 24M	21.5 ± 12.0	7.8 ± 7.7	.021
Fatty infiltration			
POD 3M			
SSC (0/1/2/3/4)	0/6/13/1/0	19/112/30/0/0	<.001
SSP (0/1/2/3/4)	0/0/4/14/2	1/28/93/36/3	<.001
ISP (0/1/2/3/4)	0/6/9/5/0	17/112/29/3/0	<.001
TM (0/1/2/3/4)	2/9/5/2/2	67/72/15/4/3	<.001
POD 6M			
SSC (0/1/2/3/4)	0/6/13/1/0	19/112/30/0/0	<.001
SSP (0/1/2/3/4)	0/0/3/13/4	2/25/95/36/3	<.001
ISP (0/1/2/3/4)	0/5/10/5/0	18/109/31/3/0	<.001
TM (0/1/2/3/4)	1/10/5/2/2	67/72/14/5/3	<.001
POD 24M			
SSC (0/1/2/3/4)	0/6/13/1/0	20/109/32/0/0	<.001
SSP (0/1/2/3/4)	0/0/3/12/5	0/28/88/42/3	<.001
ISP (0/1/2/3/4)	0/5/9/6/0	20/100/38/3/0	<.001
TM (0/1/2/3/4)	1/11/4/2/2	62/76/15/4/4/	<.001

*SSC*, subscapularis; *SSP*, supraspinatus; *ROM*, range of motion; *VAS*, visual analog scale; *CS*, Constant Shoulder; *UCLA*, University of California Los Angeles; *ISP*, infraspinatus; *IR*, internal rotation; *ER*, external rotation; *TM*, teres minor; *POD*, postoperative day.

**Table III:** Multiple logistic regression analysis results.

Variables	Odds ratio	95% CI	*P* value
Fatty infiltration of ISP	4.5	2.3-8.9	<.001

*CI*, confidence interval; *ISP*, infraspinatus.

**Table IV:** Clinical outcomes according to the severity of fatty infiltration of ISP.

Variables	Goutallier ≤2	Goutallier ≥3	*P* value
Number of shoulders	15	5	
ROM			
Forward flexion (°)			
POD 3M	153.3 ± 20.9	126 ± 48.3	.088
POD 6M	164.7 ± 5.2	148 ± 37.7	.095
POD 24M	166.7 ± 14.5	156 ± 24.1	.242
ER (°)			
POD 3M	44.7 ± 11.4	26 ± 25.1	.032
POD 6M	53 ± 9	35 ± 28.3	.038
POD 24M	55.3 ± 10.6	46 ± 34.4	.347
IR (point)			
POD 3M	4.1 ± 0.5	3.5 ± 2.5	.344
POD 6M	4.5 ± 0.9	4.2 ± 1.1	.597
POD 24M	5.2 ± 1.0	5 ± 1.1	.714
CS score (point)			
POD 3M	70.3 ± 6.3	65 ± 7.1	.331
POD 6M	74.6 ± 7.1	66.2 ± 11.8	.071
POD 24M	81.4 ± 8.6	80.6 ± 17.9	.891
UCLA score (point)			
POD 3M	23.9 ± 2.5	23 ± 1.4	.653
POD 6M	25.4 ± 2.2	23.8 ± 3.3	.232
POD 24M	28.4 ± 4.6	25.6 ± 5.1	.314

*ROM*, range of motion; *CS*, Constant Shoulder; *UCLA*, University of California Los Angeles; *ISP*, infraspinatus; *IR*, internal rotation; *ER*, external rotation; *POD*, postoperative day.

## Discussion

The main finding of this study was that ARCR using the TR technique achieved good clinical outcomes, even in cases of retear; however, the outcomes in the retear group were significantly inferior to those in the healing group. In addition, fatty infiltration of the ISP tendon was identified as an independent risk factor for retear.

The TR technique was first reported by Ostrander, who compared their novel procedure to the double-row and SB techniques using an animal model (sheep shoulders).^17^^,^^18^ The technique showed increased contact pressure on the footprint, expanded contact area, and decreased overloading on the medial row anchor. Buckup et al^1^ introduced the concept of “repositioning anchor” for the middle-row anchor used in the TR technique and described it as an anchor that is effective in preventing the retraction of the rotator cuff stump. The use of a repositioning anchor in the TR technique allows the dispersion of tension that concentrates on the medial-row anchor, which may improve the clinical outcomes of ARCR.

Despite good clinical outcomes of the TR technique being reported, limited studies have specifically examined the clinical outcomes of retear cases and the risk factors for retear of this procedure. In previous repair methods, such as the SB technique, risk factors of retear have been reported to include a larger preoperative tear size, a greater degree of fatty infiltration of the rotator cuff muscles, advanced patient age, and diabetes mellitus.^8^^,^^26^ Takahashi et al^24^ examined the relationship between early postoperative stiffness and repair integrity in patients who undergo ARCR. Their findings reported that among 155 patients, 68 (43.9%, stiff group) had postoperative stiffness at 3 months after ARCR and 87 (56.1%, nonstiff group) did not. The 12-month retear rate was patients (5.9%) in the stiff group and 15 (17.2%) in the nonstiff group, with a significantly lower retear rate in the stiff group. The study concluded that early postoperative stiffness after ARCR correlated with improved tendon healing. In this study, the retear group had greater flexion than the nonretear group at 3 months postoperatively (146.5 ± 31° in the retear group and 135.5 ± 21.9° in the nonretear group, *P* = .046). Some studies have reported that early postoperative stiffness is beneficial for rotator cuff healing.^12^^,^^13^^,^^19^ This finding is consistent with those of previous studies. It may be that patients with early postoperative stiffness experience more aggressive healing of the rotator cuff, which is related to the pathological healing process, resulting in stiffness itself. The lower retear rate may have been due to shoulder stiffness, or capsular release reducing the level of tension on rotator cuff repair.^12^ In TR technique, early postoperative ROM improvement may increase the risk of retear, suggesting the need for appropriate strategies for patients at risk of retear, such as delaying the postoperative rehabilitation schedule.

Moreover, in the retear group, the number of large and massive tear were significantly more frequent (*P* = .044), and the preoperative tear size on MRI was significantly larger in both the coronal and sagittal planes than in the nonretear group. In addition, preoperative fatty infiltration of the rotator cuff was significantly more severe in the retear group, which persisted at 3, 6, and 24 months. Multiple logistic regression analysis revealed the fatty infiltration of ISP tendon as an independent risk factor for retear of this procedure.

Management of patients with large-to-massive rotator cuff tears remains a challenge for orthopedic surgeons. Not only is it difficult to mobilize scarred retracted tendons but it is also hard to achieve a tension-free repair. Management options have included full or partial arthroscopic repair, superior capsular reconstruction, long head of biceps tendon autograft, tendon transfer, and reverse shoulder arthroplasty.^3^^,^^7^^,^^15^ However, significant retraction, muscle atrophy, and fatty infiltration can all affect repair success, and retear rates can be as high as 94%.^21^ To reduce retear for large-to-massive cuff tear repair, excessive tension should be avoided at the repaired footprint to promote optimal healing.^7^

Previous studies have reported that the ISP is the main depressor of the humeral head, and fatty infiltration of the muscle can result in proximal migration of the humerus with subacromial impingement and loss of strength in external rotation and elevation.^11^^,^^14^^,^^16^ Moreover, preoperative tear severity of the ISP and teres minor would affect the clinical outcome after rotator cuff surgery.^11^^,^^14^ Considering these studies, grading of massive rotator cuff tears based on the degree of fatty degeneration of the ISP can be useful for determining the appropriate treatment strategy for massive rotator cuff tears.^2^

In this study, preoperative fatty infiltration of the rotator cuff was significantly more severe in the retear group and persisted for up to 24 months postoperatively. In addition, multiple logistic regression analysis revealed that fatty infiltration of the ISP tendon was an independent risk factor for retear following ARCR using TR technique. The findings of this study suggest that even in ARCR using the TR technique, which generally yields favorable postoperative outcomes, cases with large-to-massive tears or severe preoperative fatty degeneration of the ISP may require additional reinforcement such as long head of bicep tendon autograft and tendon transfer.

Although we evaluated statistically significant differences in clinical outcomes between the healing and retear groups, the minimal clinically important difference for the outcome measures used in this study has not yet been clearly established for patients undergoing ARCR using the TR technique. Therefore, we were unable to determine whether the observed differences exceeded a clinically meaningful threshold. Future research is warranted to establish minimal clinically important difference values specific to this surgical technique and patient population, which will enable a more accurate interpretation of clinical significance.

Our study had several limitations. First, it was a retrospective study, which may have introduced selection bias. Second, all surgeries were performed by a single surgeon, which enhances consistency in surgical technique and postoperative management but may limit the generalizability of our findings to other surgeons or institutions. Third, the sample size was relatively small, limiting the generalizability of our findings. Fourth, the mean follow-up period was relatively short. Hence, further studies with longer follow-up periods and larger numbers of patients are needed to validate our findings.

## Conclusion

ARCR using the TR technique achieved good clinical outcomes even in cases of retears. However, the clinical outcomes were inferior to those in the healed group. Additional reinforcement may be required in cases of large-to-massive tears or severe preoperative fatty degeneration of the ISP.

## Disclaimers:

Funding: No funding was disclosed by the authors.

Conflicts of interest: The authors, their immediate families, and any research foundation with which they are affiliated have not received any financial payments or other benefits from any commercial entity related to the subject of this article.
